# Repositioning of Memantine as a Potential Novel Therapeutic Agent against Meningitic *E. coli*–Induced Pathogenicities through Disease-Associated Alpha7 Cholinergic Pathway and RNA Sequencing-Based Transcriptome Analysis of Host Inflammatory Responses

**DOI:** 10.1371/journal.pone.0121911

**Published:** 2015-05-19

**Authors:** Jing-Yi Yu, Bao Zhang, Liang Peng, Chun-Hua Wu, Hong Cao, John F. Zhong, Jill Hoffman, Sheng-He Huang

**Affiliations:** 1 Department of Microbiology, Guangdong Provincial Key Laboratory of Tropical Disease Research, School of Public Health and Tropical Medicine, Southern Medical University, Guangzhou 510515, China; 2 Saban Research Institute of Children’s Hospital Los Angeles, Department of Pediatrics, Keck School of Medicine, University of Southern California, Los Angeles, CA, 90027, United States of America; 3 Department of Clinic Laboratory, the Second Affiliated Hospital of Guangzhou Medical University, Guangzhou 510260, China; 4 Department of Pathology, Keck School of Medicine, University of Southern California, Los Angeles, CA, 90033, United States of America; 5 Department of Perio, Diagnostic Sciences & Biomedical Sciences, School of Dentistry, University of Southern California, Los Angeles, CA, 93003, United States of America; 6 Department of Pediatrics, School of Medicine, University of Southern California, Los Angeles, CA, 93003, United States of America; Shanghai Medical College, Fudan University, CHINA

## Abstract

Neonatal sepsis and meningitis (NSM) remains a leading cause worldwide of mortality and morbidity in newborn infants despite the availability of antibiotics over the last several decades. *E*. *coli* is the most common gram-negative pathogen causing NSM. Our previous studies show that α7 nicotinic receptor (α7 nAChR), an essential regulator of inflammation, plays a detrimental role in the host defense against NSM. Despite notable successes, there still exists an unmet need for new effective therapeutic approaches to treat this disease. Using the *in vitro/in vivo* models of the blood-brain barrier (BBB) and RNA-seq, we undertook a drug repositioning study to identify unknown antimicrobial activities for known drugs. We have demonstrated for the first time that memantine (MEM), a FDA-approved drug for treatment of Alzheimer’s disease, could very efficiently block *E*. *coli*-caused bacteremia and meningitis in a mouse model of NSM in a manner dependent on α7 nAChR. MEM was able to synergistically enhance the antibacterial activity of ampicillin in HBMEC infected with *E*. *coli* K1 (E44) and in neonatal mice with E44-caused bacteremia and meningitis. Differential gene expression analysis of RNA-Seq data from mouse BMEC infected with *E*. *coli* K1 showed that several E44-increased inflammatory factors, including IL33, IL18rap, MMP10 and Irs1, were significantly reduced by MEM compared to the infected cells without drug treatment. MEM could also significantly up-regulate anti-inflammatory factors, including Tnfaip3, CISH, Ptgds and Zfp36. Most interestingly, these factors may positively and negatively contribute to regulation of NF-κB, which is a hallmark feature of bacterial meningitis. Furthermore, we have demonstrated that circulating BMEC (cBMEC) are the potential novel biomarkers for NSM. MEM could significantly reduce E44-increased blood level of cBMEC in mice. Taken together, our data suggest that memantine can efficiently block host inflammatory responses to bacterial infection through modulation of both inflammatory and anti-inflammatory pathways.

## Introduction

The most common newborn deaths worldwide are neonatal infections, which currently cause about 1.6 million deaths annually in developing countries [1]. Most of these deaths are caused by bacteremia and meningitis. One of the major infectious problems in the neonatal intensive care unit is neonatal bacteremia or sepsis, which is essential for the development of bacterial meningitis [2]. This disease is associated with high mortality rates, increased medical costs and potentially poor long-term neurological sequelae [2–5]. Group B streptococcus (GBS) and *E*. *coli* are the two most common bacterial pathogens causing neonatal sepsis and meningitis (NSM) [2,6]. GBS emerged in the 1970s as a life-threatening pathogen, causing invasive infections such as sepsis and meningitis in the newborns in the US [6–8]. Intrapartum prophylaxis (IP) of GBS carriers and selective administration of antibiotics to neonates greatly reduce newborn GBS infection [6–8]. However, this has led to a major concern about whether IP use of antibiotics affects the incidence and the resistance of early-onset neonatal infection with non-GBS pathogens [6]. Currently, there has been a shift in the microbiological spectrum from GBS to *E*. *coli*, which is a leading cause of infection among neonates, particularly among those of very low birth weight (VLBW) [6,9]. *E*. *coli* is the most common cause of neonatal gram-negative bacteremia and meningitis [4–5]. Premature infants, immunocompromised hosts, and children with underlying severe gastrointestinal diseases are especially prone to *E*. *coli* sepsis and meningitis. Recent studies suggest that there is an increasing incidence of early onset *E*. *coli* infections in low birth weight and VLBW neonates and a rising frequency of ampicillin-resistant *E*. *coli* infections in preterm infants [10–11]. Widespread antibiotic use (WAU), particularly with the IP use of antimicrobial agents, may result in a rising incidence of neonatal infections with antibiotic resistance, which is an ecological and evolutionary problem stemming from the response of bacteria to antibiotics [6]. The ongoing antimicrobial resistance crisis will be certainly enhanced by WAU, leading to the increasing global incidence of infectious diseases to which we have no known reliable antimicrobial agent [12].

Despite the availability of highly bactericidal antibiotics over the last several decades, neonatal infections including bacteremia and meningitis remain a significant medical and economic problem [3–6]. There are several major limitations inherent in the conventional antimicrobial drugs, which worsen the ecological and evolutionary problem. These medicines only target microbes based on the Manichaean view of the microbe-human host relationship [13–14]. Almost all antimicrobial agents, regardless of spectrum of activity, kill both the good microbes, which may be beneficial to the host, as well as the bad germs [14]. Focusing research on individual virulence genes and the important pathogens have been the traditional approach to human infectious diseases. Another limitation of this approach is due to the inability of many drugs to reach offending intracellular organisms and to the current relative ignorance of the host antimicrobial activities. Therefore, the generation of new anti-infective agents has emerged as an unmet need in the therapeutics of microbial infection including neonatal bacteremia and meningitis. Host-directed therapeutics against pathogens may provide more effective approaches to perturbing host pathways used by pathogens in various stages of their life cycle, namely, adhesion, invasion, and growth [15,16]. Bacterial meningitis exhibits triad hallmark features (THFs): NFκB activation, pathogen penetration and leukocyte transmigration across the blood-brain barrier (BBB), which consists mainly of brain microvascular endothelial cells (BMEC) [17–19]. The most challenging issue confronting neonatal bacterial meningitis is the lack of effective therapeutic interventions against the triad features of this disease.

Our studies have shown that α7 nAChR, an essential regulator of inflammation, is critical for meningitic pathogen-induced triad features of neonatal *E*. *coli* meningitis [17–19]. Alpha7 nAChR is abundantly expressed in hippocampus, the region most vulnerable to bacterial meningitis. Distinct regulatory mechanisms and functions have been revealed for activation of α7 nAChR, which is protective in adults but deleterious in neonates [20]. Using the α7-deficient mouse cell cultures and animal model systems, we have demonstrated that α7 nAChR played a detrimental role in penetration of *E*. *coli* and polymorphonuclear neutrophil (PMN) across the BBB and in neuronal inflammation. *E*. *coli* K1 invasion and PMN transmigration across the BBB were significantly reduced in α7^-/-^ BMEC and α7^-/-^ mice. Stimulation by nicotine was abolished in the α7^-/-^ cells and animals. The same blocking effect was achieved by an α7 antagonist methyllycaconitine (MLA). Neuronal inflammation, including secretion of proinflammatory cytokines and the inflammatory response in the hippocampus, was significantly reduced in the α7-deficient mice with *E*. *coli* meningitis. Alpha7 nAChR-mediated calcium signaling in the wild-type brain endothelial cells was significantly enhanced upon exposure to nicotine and infection with the pathogen, while such cellular signaling was almost completely abolished in the α7^-/-^ cells. These findings support the notion that α7 nAChR could serve as a unique drug target for therapeutic interventions against the triad features of neonatal meningitis. In this report, using the drug repositioning approach and the *in vitro/in vivo* model systems of the BBB, we examined whether memantine, a FDA-approved drug for treatment of Alzheimer’s disease and also an α7 antagonist [21], could be used as a host-directed antimicrobial agent against the triad features of neonatal bacterial meningitis. Indeed, our new drug repositioning studies have shown that memantine could very efficiently block *E*. *coli*-induced bacteremia and meningitic infections.

## Results

### Memantine (MEM) was able to efficiently block bacterial intracellular survival in HBMEC but unable to inhibit extracellular bacterial growth

**Fig 1:**
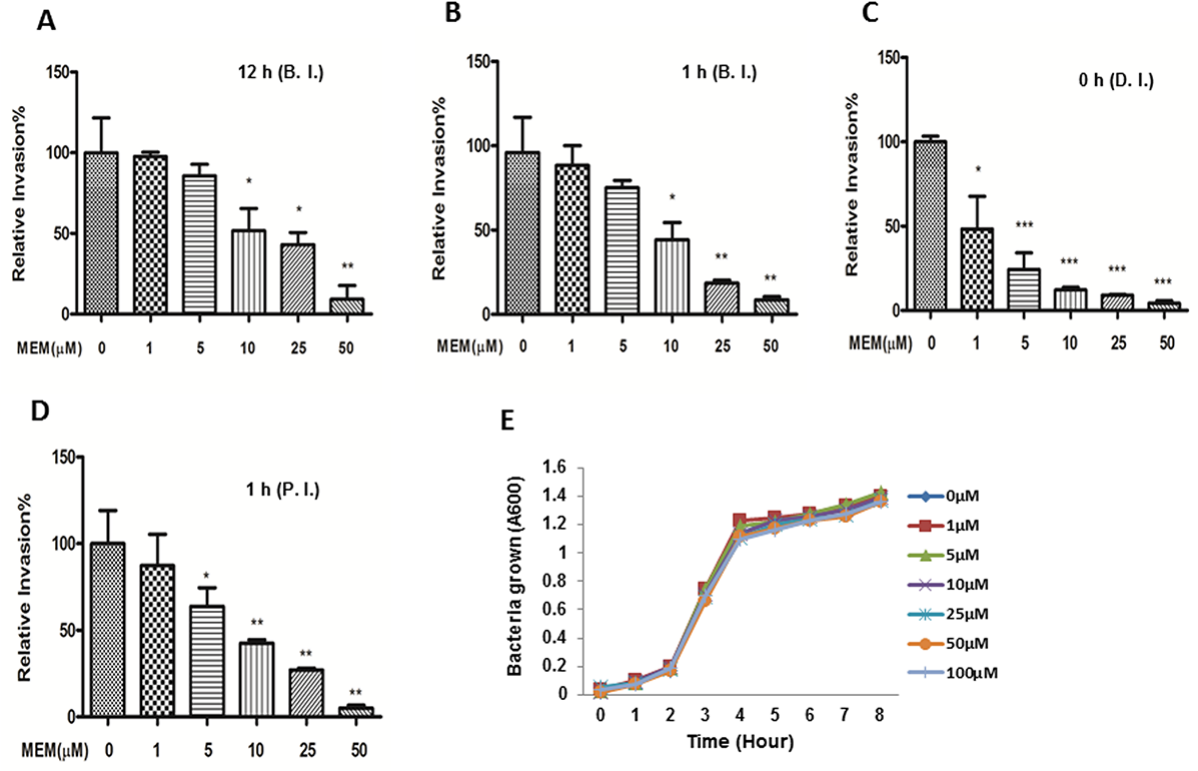
Effects of MEM on bacterial intracellular survivals of HBMEC and extracellular bacterial growth. HBMECs were incubated with various concentrations of MEM at 12 h (**A**) and 1 h (**B**) before infection (BI), 0 h during infection (**C**) (DI), or 1 h postinfection (**D**) (PI). The numbers of surviving intracellular bacteria were determined. All values represent the means of triplicate determinations. Error bars indicate standard deviations. (**E)** Effect of MEM on the growth of *E*. *coli* K1 E44 in BHI broth at various concentrations of MEM. Bacterial growth was monitored by measuring the absorbance of liquid cultures at 600 nm (A600). Similar results were obtained with the bacterial cultures grown in RPMI 1640 containing 10% FBS.

To examine whether MEM was able to block *E*. *coli* infection, we first determined the effects of this drug on bacterial internalization and survival in brain endothelial cells. HBMEC were infected with E44, a CSF isolate from a newborn baby with *E*. *coli* meningitis, and incubated with various concentrations of the drug before (Fig 1A: 12 h; Fig 1B: 1 h), during (Fig 1C) and after (Fig 1D: 1 h) bacterial infection. The numbers of surviving intracellular bacteria were determined by the invasion assay or gentamicin survival assay as described in Materials and Methods. The data demonstrate that MEM could dose-dependently inhibit bacterial intracellular survival, no matter whether the drug was present in the systems before, during and after bacterial infection. These data suggest that this drug is a potential medication used to prevent and treat meningitic infection. Because our objective was to repurpose MEM as a drug that targets host rather than bacterial functions, we examined if this agent could act directly on *E*. *coli* K1. We assessed the antimicrobial activity of MEM *in vitro* by growing bacteria in the presence of the drug (Fig 1E). Bacteria were incubated with up to 100 μM of MEM in BHI(Brain-heart infusion broth) for 1 to 8 hours at 37°C. The bacterial growth curves were determined by measuring the optical absorbance (OD_600_) at each time point. The growth rates of bacteria were compared in the presence of different concentrations of MEM at different time points. While this assay cannot distinguish between bacteriostatic and bactericidal activities of the drug, similar growth curves were obtained with or without MEM, regardless of concentration or incubation period. This result demonstrates that in the extracellular environment, MEM is unable to inhibit *E*. *coli* multiplication. Collectively, these data suggest that MEM can very efficiently block intracellular survival of *E*. *coli* K1 but has no extracellular antimicrobial activity.

### Blocking effects of MEM on α7 agonist nicotine-enhanced *E*. *coli* K1 invasion and PMN transmigration across HBMEC

**Fig 2:**
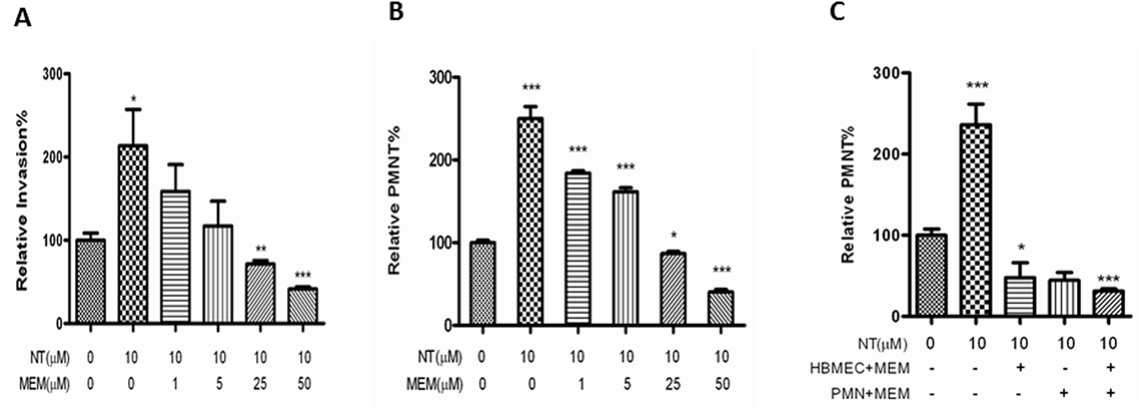
Effects of MEM-mediated blockages of α7 nAChR on nicotine-enhanced E44 invasion and leukocyte transmigration. E44 invasion (**A**) and leukocyte (PMN) transmigration (**B-C**) across HBMEC after exposure to nicotine (NT). **(A-B)** Effects of different doses of MEM (1 h incubation) on E44 invasion (**A**) and PMN transmigration (**B**) across HBMEC treated with (10 μM NT for 48 h) and without NT. **(C)** Effect of MEM treatment of either HBMEC or PMN on NT-enhanced PMN transmigration. HBMEC were pre-exposed to 10 μM NT for 48 h, and then HBMEC and PMN were treated with MEM for 1 hr prior to the leukocyte transmigration assay. In all treatments, HBMEC without any treatment was taken as a control. All results are expressed as relative invasion and PMN transmigration compared to the corresponding controls without treatments. All invasion and PMN transmigration assays were performed in triplicate wells. Bar graphs show the means ± SD of triplicate samples. Significant differences with regard to the controls are marked by asterisks (*P<0.05; ***P<0.001).

To further determine if MEM could serve as an α7 antagonist to inhibit nicotine-enhanced pathogenicities of meningitic *E*. *coli* K1, we examined blocking effects of this drug on pathogen penetration and PMN transmigration across HBMEC treated with and without nicotine. To mimic the concentrations of nicotine measured in the serum of human active and passive smokers [22], HBMEC were exposed to low doses of nicotine (10 μM) for 48 h, and then treated with different doses (1–50 μM) of the α7 antagonist MEM. The cells were subjected to bacterial invasion and PMN transmigration assays. The result indicated that MEM was able to block *E*. *coli* invasion of HBMEC treated with nicotine in a dose-dependent manner (Fig 2A). In order to exclude the possibility that the leukocyte migration elicited was due to destruction of HBMEC, the integrity of the monolayer was inspected by microscopy. As indicated in Fig 2B, MEM was able to significantly inhibit nicotine-enhanced PMN transmigration across the HBMEC monolayer in a dose-dependent manner. MEM-mediated blocking effects were observed upon treatment of either cell type alone or both (Fig 2C), suggesting that α7 nAChR expression on both leukocytes and HBMEC is required for nicotine-enhanced PMN transmigration *in vitro*. These findings were consistent with the result of chemical blockage by the α7 antagonist MLA [18], suggesting that α7 nAChR on BMEC and PMN is required for leukocyte transmigration. Taken together, these studies suggest that α7 nAChR contributes to MEM-mediated blocking effects on nicotine-enhanced bacterial invasion and PMN transmigration across HBMEC.

### Comparative analysis of the effects of NMDAR and α7 nAChR inhibitors on bacterial intracellular survivals of HBMEC

**Fig 3:**
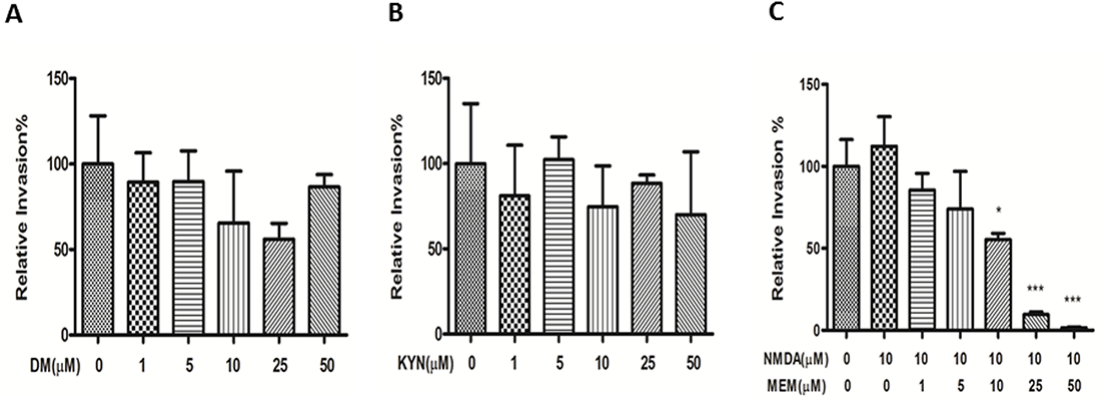
Comparative analysis of the effect of MEM, NMDA and two NMDAR antagonists (DM and Kyn) on bacterial intracellular survival. HBMECs were incubated with various concentrations of DM (**A**) and Kyn (**B**) 24 h before adding bacteria. (**C**) Effect of NMDA (10 μM) on bacterial intracellular survivals of HBMEC. All values are presented as relative invasion %. All invasion assays were performed in triplicate wells. Bar graphs show the means ± SD of triplicate samples. Significant differences between the treatment and the control groups are marked by asterisks (*P<0.05; ***P<0.001).

MEM has been shown to be the dual inhibitor of α7 nAChR and n-methyl-D-aspartate receptors (NMDARs), while it blocks α7 nAChR more potently than NMDARs in rat hippocampal neurons [21]. To determine whether blockage of NMDARs could affect intracellular survival of meningitic *E*. *coli* K1 in a manner similar to the inhibition of α7 nAChR, we next performed comparative analysis of the effects of NMDAR and α7 nAChR inhibitors on bacterial intracellular survivals of HBMEC. The effects of MEM, NMDA (NMDA agonist) and two NMDAR antagonists, kynurenic acid (Kyn) [23] and dextromethorphan (DM) [24], were compared using the gentamicin survival assay. As shown in Fig 3, DM and Kyn (Fig 3A and 3B) could not significantly block bacterial intracellular survivals of HBMEC and no dose-dependent effects were observed for these two drugs when compared to that of MEM. Furthermore, no significant stimulating effect was observed with the NMDAR agonist NMDA at 10 μM that is the same dosage of the α7 agonist nicotine, which is able to significantly enhance *E*. *coli* K1 internalization of HBMEC (Fig 3C). These findings demonstrate that MEM-mediated blockage of bacterial intracellular survivals mainly depends on α7 nAChR.

### Blocking effects of MEM on bacteremia, meningitis, BBB injury and inflammatory signaling

**Fig 4:**
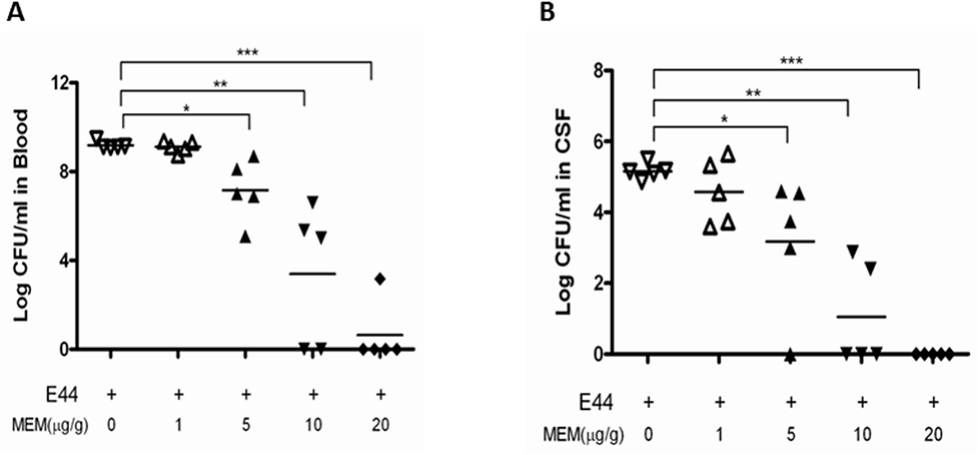
Dose-dependent blockage of bacteremia and meningitis. C57BL/6J mice (10days) were injected (i.p.) twice with various concentrations (0–20 mg/kg) of MEM at 12 h before and at the time of bacteria inoculation (2×10^5^ CFU of E44). Bacteremia and meningitis in mice were evaluated at 18h after infection. The numbers of surviving bacteria in blood (**A**) and CSF (**B**) were determined. Neonatal mice were divided into 5 groups (5 pups/group). All values represent the means of determinations. Each experiment was performed three times. *P<0.05, **P<0.01, ***P<0.001.

**Fig 5:**
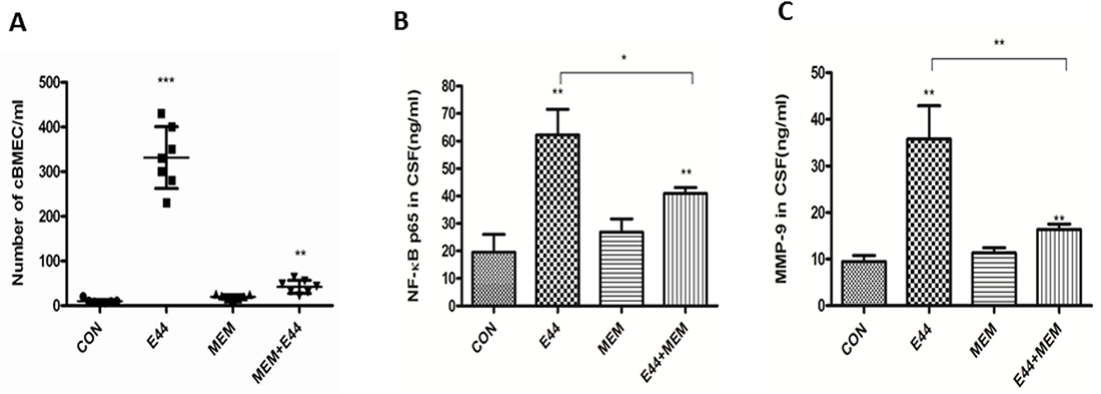
Blocking effects of MEM on BBB injury and inflammatory responses. **(A)** Peripheral blood concentration of cBMEC in mice treated with or without MEM. **(B)** Magnitude of NF-κB (p65) activation in mice treated with or without MEM. **(C)** Cerebrospinal fluid (CSF) concentration of MMP-9 in mice treated with or without MEM. Neonatal mice were divided into 4 groups (7 pups/group). Each experiment was performed three times. *P<0.05, **P<0.01, ***P<0.001.

To further determine the biological relevance of the *in vitro* assays, the efficacy of MEM on neonatal *E*. *coli* K1 meningitis was tested in the mouse model, as described in Methods and Materials. First, we investigated whether MEM could dose-dependently block bacteremia and meningitis in neonatal mice (10 day-old). Mice were treated with different doses (1–20 mg/kg) of MEM, beginning 12 h prior to infection and continuing for the experiment. The animals were infected with E44 (2x10^5^ CFU). Our data show that this drug could dose-dependently block bacteremia (Fig 4A) and meningitis (Fig 4B). MEM reduced median bacterial loads in blood and CSF by 2.3log_10_ to 7.7log_10_ CFU/ml in response to the drug treatment at doses of 1 to 20 mg/kg. The blocking effects in this range are statistically significant (p values between 0.001 and 0.05). Both bacteremia and meningitis are almost completely blocked by MEM at the dose of 20 mg/ml. In the second experiment, wild-type neonatal (10 day-old) mice were divided into four groups. They were intraperitoneally injected with or without E44 (2x10^5^ CFU) and treated with or without MEM (20 mg/kg) at 12 h before bacterial inoculation. As shown in Table 1, MEM was able to significantly block *E*. *coli* bacteremia (*P*<0.01) and bacterial entry into CSF (meningitis) (*P*<0.01). MEM could also significantly reduce the blood level of the BBB cellular biomarker cBMEC (Fig 5A), the magnitude of NF-κB activation (p65 in CSF)(Fig 5B) and the CSF concentration of MMP-9 (Fig 5C) when compared to the controls without drug treatment. These results suggest that MEM could decrease the host susceptibility to *E*. *coli* K1 infection (reduced bacteremia and meningitis), CNS inflammatory response (reduced p65 and MMP-9 in CSF) and BBB injury (reduced cBMEC in blood).

**Table 1:** The rates of bacteremia, and meningitis in C57BL/6J mice (10 days) after receiving Memantine and E44.

Treatment	No. of animals	Bacteremia (log CFU/ml of blood, mean ± SD)	No.(%) of pups with bacteremia	Meningitis (log CFU/ml of CSF, mean ± SD)	No.(%) of pups with meningitis
PBS	7	0	0(0)	0	0(0)
E44	7	9.02±0.07	7(100)^a^	4.99±0.21	7(100)^a^
MEM	7	0	0(0)	0	0(0)
MEM+E44	7	0.45±0.00	1(14.4)	0	0(0)

Chi-Square test,

^a^ *p* < 0.01 vs. MEM+E44 group;

CFU: Colony-forming unit;

CSF: Cerebrospinal fluid;

SD: Standard deviation.

### MEM was able to synergistically enhance the antibacterial activity of ampicillin in HBMEC infected with meningitic *E*. *coli* K1 (E44) and in neonatal mice with bacteremia and meningitis

**Fig 6:**
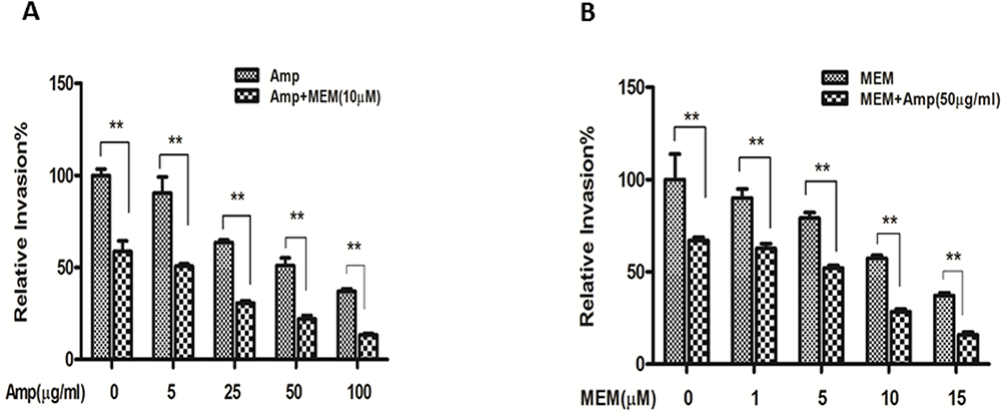
MEM potentiates intracellular killing of *E*. *coli* K1 in HBMEC with a combination of Amp. **A**: Five combination settings of HBMEC were tested with the same concentration (10 μM) of MEM and different concentrations of Amp (0, 5, 25, 50 and 100 μg/ml). **B**: Five combination settings of cells were treated with the same amount (50 μg/ml) of Amp and different concentrations of MEM (0, 1, 5, 10, and 15 μM). The intracellular killing activity with a combination of drugs was significantly higher (**P<0.001) than that of the treatment with one drug alone.

**Fig 7:**
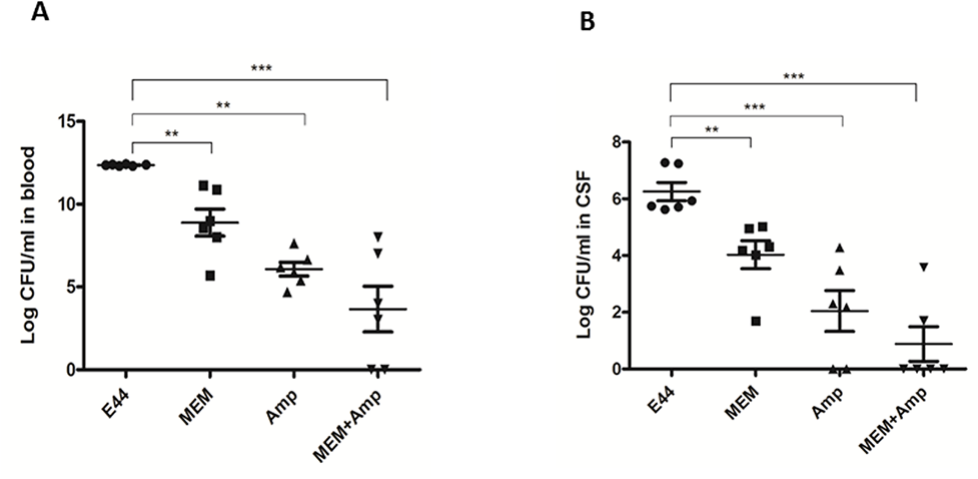
MEM and Amp synergistically block the magnitude of bacteremia and meningitis. C57BL/6J mice (10days) were injected (i.p.) with MEM (20 mg/ kg) and Amp (20 mg/ kg) alone, or MEM in combination with Amp. The treatment was started at 6 h after bacterial infection (2×10^5^ CFU of E44). Bacteremia and meningitis in mice were evaluated at 14h after drug treatment. The bacterial loads in blood (**A**) and CSF (**B**) were determined. Neonatal mice were divided into 5 groups (n = 6). All values represent the means of determinations. Each experiment was performed three times. **P<0.01, ***P<0.001.

In order to further define the mechanisms of MEM-mediated intracellular blockage of *E*. *coli* K1infection, we have examined *in vitro* and *in vivo* whether a combination of MEM and an antibiotic is superior to either medication alone in the treatment of bacterial infection. We determined the effects of MEM in conjunction with ampicillin (Amp), one of the most common antibiotics used for treatment of neonatal sepsis caused by Group B streptococcus and susceptible *E*. *coli* strains [25]. HBMECs in the 24-well plates were infected with E44 for 1 h at 37°C and then incubated with gentamicin (10 mg/ml) for 1 h to eliminate extracellular bacteria. Under this condition gentamicin does not affect intracellular growth of E44 (data not shown). HBMECs were treated with Amp (5 to 100 μg/ml) or MEM (1 to 15 μM) alone, versus MEM in combination with Amp at the concentrations indicated. Intracellular survival of E44 was determined at 1 h. For these experiments, the concentrations of drugs chosen only partially inhibited bacterial growth. Combination treatment of MEM with Amp (Fig 6A and 6B) showed a stronger inhibitory effect on E44 infection. Determination of a synergistic, additive or antagonistic effect of MEM and Amp combination was performed according to the median effect principle using the CalcuSyn Software (Biosoft). The CI values for the combination treatment of MEM and Amp were less than 1, suggesting that the drug combination is highly synergistic. Together, these data suggest that the combination of MEM and Amp produced a synergistic reduction in the survival of meningitic *E*. *coli* K1 in HBMEC. Furthermore, the biological relevance of the *in vitro* study has been confirmed in the mouse model of NSM. The therapeutic efficacy of MEM and Amp alone and in conjunction was investigated in terms of reduction in the magnitude of bacteremia and the number of bacteria in CSF of neonatal mice infected with E44. The treatment was started after 6 h of bacterial inoculation and continued for 14 h. The adjunct therapy of 20 mg MEM with 20 mg Amp/kg body weight was found to be synergistic (Fig 7A and 7B).

### Identification of new potential inflammatory and anti-inflammatory factors in mouse BMEC infected with meningitic *E*. *coli* K1- and treated with MEM through RNA sequencing analysis

**Fig 8:**
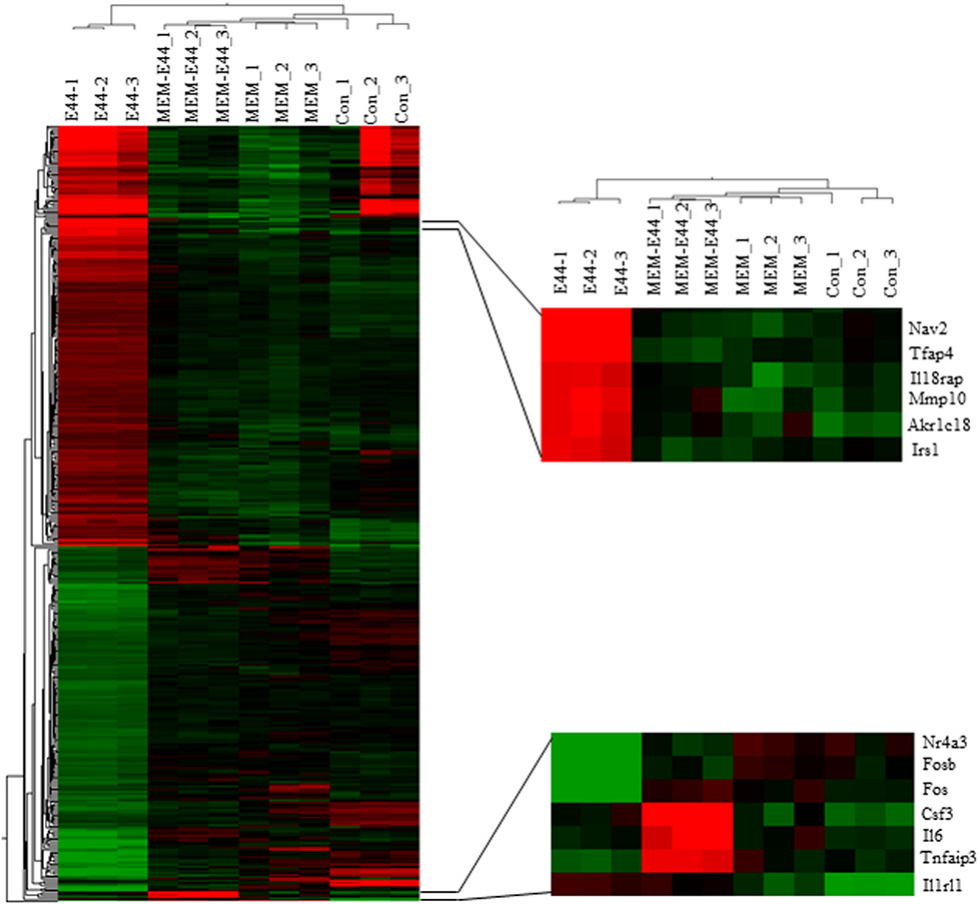
Transcriptome profile differences between the MEM-treated (MEM; E44+MEM) and untreated control (CON; E44) groups. The heat map diagram shows the patterns of differentially expressed genes (DEG) within and between biological replicates. MEM-1, MEM-2, MEM-3, CON-1, CON-2 and CON-3 represent biological replicates of uninfected MBMEC, while E44-1, E44-2, E44-3, MEM-E44-1, MEM-E44-2 and MEM-E44-3 represent biological replicates of E44-infected MBMEC. The color red on the heat map indicates upregulation during infection, while the color green indicates downregulation during infection.

**Fig 9:**
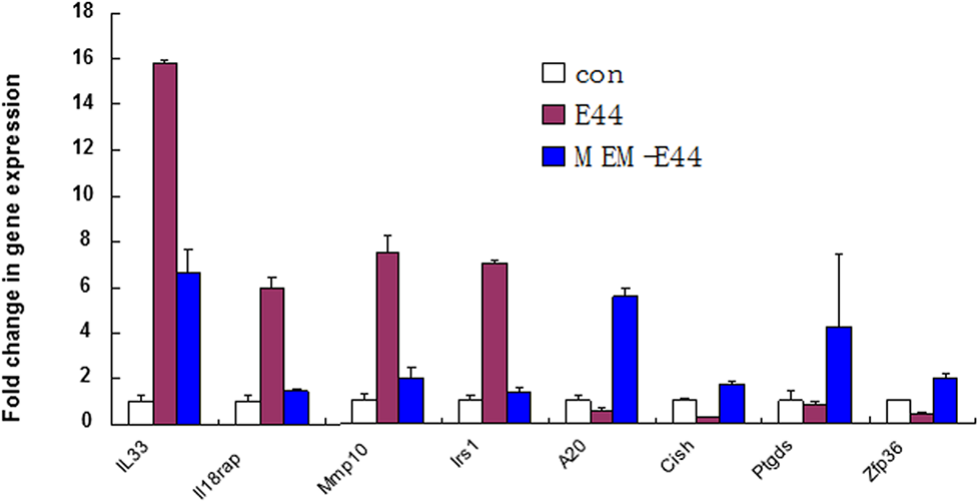
RNA-seq analysis of BMEC infected with *E*. *coli* and treated with MEM. Mouse BMECs (triplet) were treated with E44 (MOI: 10:1 for 1.5 h), PBS (Con), MEM (50 μM 12 h) and E44/MEM. Twelve RNA samples were prepared and sent to BGI America for RNA sequencing. Four inflammatory (IL-33, IL-18rap, MMP10 and Irs1 and anti-inflammatory (A20, CISH, Ptgds and ZFP36) genes are significantly down- and up-regulated, respectively, in E44-infected cells treated with MEM when compared to the infected BMEC without treatment.

To further elucidate the underlying mechanisms responsible for the blocking effects of MEM on *E*. *coli* bacteremia and meningitis, global gene expression analysis of mouse BMEC (MBMEC) infected with E44 and treated with MEM was carried out using RNA-seq, which was performed by BGI Americas [26]. The gene lists were further analyzed with the DAVID Functional Annotation Clustering Tool and PANTHER Classification System to identify significantly over-represented ontology categories and molecular pathways [27]. A comparison of the responses of MBMEC to *E*. *coli* K1 infection and MEM treatment revealed distinct gene expression profiles. Heat map (Fig 8) illustrates the general gene expression pattern in relation to the two main factors—*E*. *coli* K1 infection and MEM treatment. The heat map indicates a good clustering of samples according to whether the infection was treated or untreated. There is a clear distinction between these two groups and the gene expression profiles were able to discriminate between the two main groups, with and without MEM. Altogether 978 genes out of the analyzed 17,689 genes in the *E*. *coli* K1-infected cells treated with MEM showed statistically significant differential expression patterns (*P*<0.05 or 0.01). To identify genes important for host inflammatory and anti-inflammatory responses, genes that are significantly up- or down-regulated in E44-infected MBMEC treated with or without MEM were selected and analyzed. Several E44-increased inflammatory factors, including IL33, IL18rap, MMP10 and Irs1, were significantly reduced by MEM compared to the infected cells without drug treatment (Fig 9). MEM could also significantly up-regulate anti-inflammatory factors, including Tnfaip3, CISH, Ptgds and Zfp36 [28–32]. Most interestingly, these factors may positively (e.g., IL33 and IL18rap) and negatively contribute to regulation of NF-κB, which is a hallmark feature of bacterial meningitis. MEM could also significantly reduce E44-increased blood level of cBMEC in mice (Fig 5A). These novel findings suggest that MEM can efficiently block host proinflammatory pathways meanwhile enhancing anti-inflammatory responses to bacterial infection.

### Discussion

Recently, the concept of host-directed antimicrobial therapeutics against intracellular pathogens that hijack cellular signaling pathways and deploy host defense mechanisms has received widespread attention in the scientific community [16,33,34]. Shifting from the single to the two-way paradigm of host-microbe interactions [14], targeting of host factors that are essential for intracellular survival of pathogens with a small-molecule agent or repositioning of a FDA-approved drug offers a novel therapeutic strategy for infectious diseases. Using the *in vitro* (BMEC) and *in vivo* (neonatal mice) models of the BBB, we present novel findings that provide proof of principle of the feasibility of treating meningitic *E*. *coli* K1 infection by targeting the host receptor α7 nAChR that is an essential regulator of inflammation manipulated by bacterial pathogens. Our findings show that the FDA-approved drug MEM is a potent inhibitor of the intracellular survival of meningitic *E*. *coli* K1 in BMEC. It induces antimicrobial effects with a submicromolar IC_50_ and, importantly, in the absence of host cell toxicity and without direct inhibitory effect on bacterial growth. Because there is no direct activity against the pathogens, it is most likely that microbial resistance to the host-targeted drugs like MEM is less likely to arise than the conventional antibiotics. Moreover, this drug has been used to treat Alzheimer’s disease over 30 years showing a favorable safety and tolerability profile when used as monotherapy or in combination with other agents [35]. Animal studies also demonstrate the relative safety of MEM at neuroprotective doses in the immature rodent brain [36,37]. Our *in vivo* evaluations of MEM in the mouse model of neonatal bacteremia and meningitis revealed that continuous treatment with doses of up to 20mg/kg/day was well tolerated. These findings suggest that the favorable safety and tolerability profile associated with this low antibacterial dose of MEM may provide a promising therapeutic intervention in neonatal bacteremia and meningitis.

In this report, we demonstrate that MEM could exhibit antimicrobial activity on intracellular bacterial survival at both the early (bacteremia) and late (meningitis) stages of *E*. *coli* K1 infection and that each of these stages has a distinct role in the pathogenesis and therapeutics of this disease. Studies in humans and experimental animals have shown that there is a relationship between the magnitude of bacteremia and the development of meningitis. High-grade bacteremia precedes meningitis and then bacteria invade from the blood stream to the central nervous system (CNS) [5,38]. Our data indicate that the inhibition of bacteremia plays a major role in the early stage of MEM’s antibacterial effect, suggesting that this drug can hinder pathogen-BBB interaction and therefore prevent disease progression. As shown in the animal experiments, the degree of bacteremia inhibition is correlated with the development of meningitis. MEM at the dose of 20 mg/kg could almost completely block bacteremia and meningitis. Furthermore, this drug is shown to be highly effective in blocking of bacterial invasion, NF-κB activation and leukocyte transmigration that occur at the BBB, which are the triad hallmark features of bacterial meningitis [17–19,39]. MEM could significantly reduce host factors contributing to pathogen invasion (MMP-9 in CSF), NF-κB activation (p65 in CSF) and BBB injury (cBMEC in blood). Together, these findings suggest that MEM is able to either efficiently block the early stage infection (bacteremia) or block the transition from early to the development of the triad features of bacterial meningitis, which make good drug target sites.

MEM, a FDA-approved drug for treatment of Alzheimer’s disease (AD), is a dual inhibitor of NMDARs and α7 nAChRs [21]. Although the cause of AD remains unclear, the proposal of the cholinergic hypothesis of memory impairment has been accepted for the drug development of this disease since 1984 [40]. NMDAR activities also contribute to the insufficiency of the AD brain [21]. Since the 1980s, despite the evaluation of numerous potential treatments in clinical trials, the low-affinity, noncompetitive NMDAR antagonist MEM has been approved as one of the major drugs for treatment of AD. It has shown sufficient safety and efficacy worldwide over the past 30 years [40]. It has been demonstrated that resembling nicotinic antagonists, MEM affects cognition in rats and healthy human subjects. Furthermore, there is evidence that MEM inhibits α7 nAChR (IC_50_ = 0.34 μM) more potently than NMDA receptors (IC_50_ = 5.1 μM) in rat hippcampal neurons [21]. Concurring with these findings, our data show that MEM-mediated blockage of meningitic *E*. *coli* K1 infection mainly depends on the α7 nAChR cholinergic pathway. Clinical observations in humans suggest that an increased incidence of bacterial meningitis is associated with exposure to second hand tobacco smoke containing nicotine, the α7 agonist that enhances α7 nAChR activation [41]. Our animal studies show that nicotine is able to significantly enhance *E*. *coli* K1 invasion and leukocyte transmigration across the BBB in vitro and in vivo in a manner dependent on α7 nAChR [18]. In this report, we demonstrated that MEM could dose-dependently block nicotine-enhanced pathogenicities of *E*. *coli* K1 at the low dose (10 μM) that mimics the concentrations of nicotine measured in the serum of human active and passive smokers [22]. However, NMDA, a NMDAR agonist, did not show significant stimulating effects at the same concentration. Moreover, the NMDAR antagonists, kynurenic acid [23] and dextromethorphan [24] could not significantly block *E*. *coli* K1 invasion of HBMEC when compared to the blocking effects mediated MEM at the same dose range. Together, these findings indicate that α7 nAChR plays an important role in the MEM’s efficacy of blocking *E*. *coli* bacteremia and meningitis. Because α7 nAChR and NMDARs are both ligand-gated ion channels permeable to Ca2+ and Na+ there may be a cross-talk between the two pathways [42]. It remains to be determined whether their cross-talk plays a role in the drug activity of MEM for treatment of neonatal sepsis and meningitis.

The inflammatory response to bacterial invasion as a systemic reaction has been studied using both cellular and molecular approaches. It has been recognized that the host’s response to bacteremia or sepsis, and likely also bacterial meningitis, represents a pathological inflammatory response and therefore causes more damage than the pathogen itself. This inflammatory response is triggered by changes in transcriptomes and whole genome expression profiling [43]. In order to further determine how MEM could block meningitic infection, we identified up- or down-regulated transcriptional pathways in MBMEC infected with meningitic *E*. *coli* K1 and treated with MEM using RNA-seq transcriptional profiling combined with pathway analysis. Several E44-increased inflammatory factors, including IL33, IL18rap, MMP10 and Irs1, were significantly reduced by MEM compared to the infected cells without drug treatment. IL-33 and IL-18 are cytokine belonging to the IL-1 family [32] The IL18r1 and IL18rap genes code for the components of the heterodimeric IL18 receptor [32]. Signaling cascades triggered by IL-18 and IL-33 activate MAPKs and NF-kappa B, leading to the expression of proinflammatory cytokines, chemokines, and secondary mediators of the inflammatory response. IL18rap, MMP10 and Irs1 have been recently shown to play a role in the pathogenesis of CNS inflammation [43–45]. MEM could also significantly up-regulate anti-inflammatory factors, including Tnfaip3, CISH and Zfp36 [28–32]. Tnfaip3 and CISH are important for host defense against infectious diseases and involved in negative feedback regulation of the tumor promoting effects [28,30,46]. ZFP36 is able to suppress inflammatory response in endothelial cells via both transcriptional and posttranscriptional mechanisms [31]. Most interestingly, these factors may positively (e.g., IL33 and IL18rap) and negatively (Tnfaip3, CISH and Zfp36) contribute to regulation of NF-κB, which is a hallmark feature of bacterial meningitis. MEM could also significantly reduce E44-increased blood level of cBMEC in mice. These novel findings suggest that MEM can efficiently block host inflammatory responses to bacterial infection through modulation of both proinflammatory and anti-inflammatory pathways. It remains to be determined how these factors contribute to the pathogenesis and therapeutics of neonatal sepsis and meningitis.

In conclusion, MEM represents a promising host-directed antimicrobial agent that can be developed as a novel therapeutic intervention targeting host cells for the treatment of neonatal sepsis and bacterial meningitis. Additionally, our data suggest that MEM may synergistically enhance the antimicrobial activity of the conventional antibiotics. Because MEM targets host receptors such as α7 nAChR, not bacterial factors, it is more likely to reduce the risk of development of antimicrobial resistance compared to the conventional antibiotics. Moreover, this drug can efficiently block NSM at both the early (bacteremia/sepsis) and late (meningitis) stages of this disease.

## Methods and Materials

### Ethics statement

All research involving human participants has been approved by the Institutional Review Board (IRB) of Children’s Hospital Los Angeles (CHLA). Human polymorphonuclear leukocytes (PMNs) were isolated from heparin anticoagulated, human peripheral venous blood of healthy adult volunteers using the standard Ficoll-Hypaque method in accordance with the protocol approved by the CHLA Committee on Clinical Investigations (CCI), which is the IRB for Human Subjects at Saban Research Institute of CHLA. Human brain microvascular endothelial cells (HBMEC) were isolated in accordance with the protocol approved by the CHLA Committee on Clinical Investigations (CCI), which is the IRB for Human Subjects at Saban Research Institute of CHLA. This protocol has been granted a waiver of informed or signed consent per 45 CFR 46.116(d) and a waiver of HIPAA authorization per the Privacy Rule (45 CFR Part 160 and Subparts A and E of Part 164). No minors/children participants were involved in our studies. The animal study was performed in strict accordance with the recommendations in the Guide for the Care and Use of Laboratory Animals of the National Institutes of Health. Our protocols were approved by the Institutional Animal Care and Use Committee (IACUC) of The Saban Research Institute of CHLA (Permit number: A3276-01). All surgery was performed under anesthesia with ketamine and lidocaine, and all efforts were made to minimize suffering.

### Chemicals and reagent

Nicotine tartrate (NT), n-methyl-D-aspartate (NMDA), Ampicillin (Amp), kynurenic acid (Kyn) and dextromethorphan (DM) were purchased from Sigma-Aldrich (St. Louis, MO). Memantine (hydrochloride) was purchased from Cayman(Ann Arbor, MI). Dynabeads M-450 Tosylactivated was purchased from Invitrogen (Carlsbad, CA). Ulex europaeus I (UEA I) lectin and mounting medium with DAPI were purchased from Vector (Buringame, CA). All primary antibodies (Ab) were purchased from the commercial sources: a rabbit anti-MSFD2 Ab (sc-135305), a goat anti-MMP-9 Ab (sc-6841) and a rabbit anti-NF-κB p65 (sc-109) from Santa CruzBiotechnology (Santa Cruz, CA); an anti- CD146 Ab PE-conjugated (12-1469-41) from eBiosciences, (San Diego, CA). Transwell filters (3 μm pore size, 6.5 mm diameter) was purchased from BD Biosciences (San Jose, CA).

### Mice

The C57BL/6J background (B6.129S7-Chrna7^tm1Bay^/J) were purchased from Jackson Laboratory (Bar Harbor, ME). The animals were used in breeding at 8 weeks of age for optimum reproductive performance. The average litter size for neonatal mice was 6–8. Age- and sex-matched mice were used in all experiments. All experiments were approved by the Animal Care and Use Committee of Childrens Hospital Los Angeles Saban Research Institute.

### Isolation and purification of mouse brain microvascular endothelial cells

Mouse BMEC (MBMEC) were isolated from blood and brain tissues with Ulex europaeus I (UEA I) lectin-coated Dynabeads as described previously [47]. The beads were prepared according to the manufacturer’s instructions (Invitrogen) and resuspended in Hanks' balanced salt solution (HBSS, Invitrogen Corp., Carlsbad, CA, USA) plus 5% fetal calf serum (HBSS+5%FCS) to a final concentration of 4xl0^8^ beads/ml. The MBMEC and cBMEC were prepared as described previously [18,48]. Briefly, microvascular capillaries from brain tissues and endothelial cells from blood were isolated by absorption to Ulex-coated beads. The cells were positive for CD146 [48], demonstrating their endothelial origin, and also expressed MFSD2a [49], indicating their brain origin. For the cBMEC assays, the cells were transferred to glass splices to by cytospin for staining and counting under a fluorescence microscope. Total ECs or CECs (CD146+/DAPI+) and cBMECs (CD146+/Mfsd2a+/DAPI+) were identified based on their Mfsd2a [49] (brain marker)^+^/CD146 (EC marker)^+^/DAPI (nuclei)^+^phenotypes.

### Bacterial strains, culture conditions, plasmids and media

*E*. *coli* strain RS218 (018:K1: H7) is a clinical isolate from the CSF of a neonate with meningitis [18]. E44 is a rifampin-resistant strain derived from RS218, which has been characterized. Both RS218 and E44 have the same virulence phenotypes. Bacteria were cultured in Brain-heart infusion (BHI) broth and stored in BHI with 20% glycerol at -70°C. Growth conditions affecting *E*. *coli* invasion were examined by using E44 grown in L broth overnight without agitation, unless otherwise specified. The effect of MEM was tested by diluting overnight bacterial cultures in LB medium containing different concentration of the drug (1–100 μM) to 0.1 optical density unit at 600 nm, and then grown for up to 8 h. Growth rates of E44 were determined at different time intervals.

### Invasion assay

To test the effects of drugs on *E*. *coli* internalization, invasion assays were performed as described previously [18, 50]. Briefly, after exposure to drugs, cell cultures were examined under a microscope. Confluent cells in 24-well plates were incubated with 1×10^7^ *E*. *coli* (multiplicity of infection of 100) in experimental medium (1: 1 mixture of M199: Ham’s F-12 containing 5% heat-inactivated fetal bovine serum) for 90 min at 37°C. The monolayers were washed with HBSS (Hank’s Balanced Salk Solution) and then incubated in experimental medium containing gentamicin (100 μg/ml) for 1 h to kill extracellular bacteria. The monolayers were washed again and lysed with 0.5% Triton X-100. The released intracellular bacteria were enumerated by plating on L broth agar plates. The actual inoculum size was determined by colony plate count for every experiment. Each assay was conducted in triplicate and repeated at least three times. Bacterial viability was not affected by 0.5% Triton X-100 treatment. The MIC of gentamicin for all strains used was 1 μg/ml. Cell viability was routinely verified by Trypan blue staining assay. Results were expressed as relative invasion (percentage of invasion in comparison to that of untreated BMEC). MEM, NMDA, nicotine, kynurenic acid and dextromethorphan (DM) were used to examine their effects on *E*. *coli* invasion. All drugs were present throughout the invasion experiments until the medium was replaced with experimental medium (EM) containing gentamicin. Their effects on *E*. *coli* and BMEC was examined by bacterial colony counting and trypan blue staining methods, respectively.

### PMN transmigration

PMNs were isolated according to standard techniques from heparin anticoagulated venous blood [17]. Leukocyte transmigration assays were performed as described previously [18] with modification. To test the effects of nicotine on PMN transmigration, BMEC were subcultured on transwell filters (3.0-μm pore size, 6.5mm diameter) and exposed to nicotine as described above. The confluence of the monolayer was confirmed by light microscopy before the start of the assay. E44 (10^5^ CFU/ml) was added to the lower chambers and incubated for 2 h. Then, PMN (1×10^6^ cells) were added to the upper chamber and allowed to migrate over for 4 h. At the end of the incubation, migrated PMN cells were collected from the lower chamber and counted as described previously [18]. All experiments were performed with triplicate wells. For inhibitions of PMN transmigration, cells were incubated with inhibitors for 1 h before E44 stimulation. All inhibitors were present throughout the experiment. The integrity of BMEC monolayers on Transwell filters was examined before and after PMN migration.

### Mouse model of *E*. *coli* bacteremia and meningitis

For the study on the therapeutic efficacy of MEM, mice (5–8 mice each group) were treated with or without the drug. MEM treatment started from 12 hours by intraperitoneal injection (20mg/kg body weight) before bacterial inoculation. At 10 days of age, pups received *E*. *coli* K1 strain E44 (2×10^5^ CFU) by intraperitoneal injection. Eighteen hours after *E*. *coli* inoculation, animals were anaesthetized with ketamine and lidocaine, and blood samples were collected from heart puncture for bacterial culture using L broth plates containing rifampin (50 μg/ml). After perfusion from heart puncture with 20 ml PBS [18], the skull was opened. CSF samples were collected by washing the brain tissues with 100 μl of PBS, and then by washing the cerebral ventricles and cranial cavity with another 100 μl of PBS as described previously [18]. CSF samples containing more than 10 erythrocytes per μl were discarded as contaminated samples [17, 51]. For bacteria counting in CSF, 20 μl CSF samples were taken and diluted for bacterial culture with L broth plates containing rifampin. MMP-9 and NF-κB p65 in CSF samples were determined using the ELISA kit from Bethyl laboratories (Montgomery, TX) according to the manufacturer.

### *E*. *coli* K1 infection and drug treatment of MBMEC

Host cells were grown as monolayers in dishes until 90% confluent. Monolayers were infected with E44 in 3.5 mL experimental medium for an MOI of 10:1 as previously described [18]. A matching number of MBMEC monolayers were also mock-infected using uninfected cell lysates. Each treatment was incubated for 1.5 h and subsequently washed twice with PBS to remove dead or nonviable cells. Three mL fresh medium (DMEM+2% FBS, 10 mg/ml gentamycin) was added and cell monolayers incubated for 1.5 h at 37°C with 5% CO2. Cells were harvested postinfection for RNA extraction.

### Total RNA purification

Prior to RNA extraction, work surfaces and equipment were specially treated to inactivate RNases. RNA was extracted from twelve samples composed of 3 uninfected MBMEC (control), 3 uninfected MBMEC with MEM, 3 infected MBMEC (E44), and 3 infected MBMEC treated with MEM (E44 + MEM). All samples were collected after 3 h postinfection. Cell RNA was extracted with TRIzol reagent following manufacturer’s instructions (Invitrogen, Carlsbad, CA) and treated with Turbo DNase (Ambion, Austin, TX) at 37°C for 30 min. Turbo DNase was inactivated using phenol/chloroform extraction. RNA purity was assessed by measuring the 260/280 ratio with the Nanodrop ND-1000 (Nanodrop Products, Willimington, DE). RNA samples were stored at −80°C until further processing.

### Sample processing and RNA-Seq

In order to ensure RNA integrity and purity, all samples were quantified using the RiboGreen assay per manufacturer’s instructions (Invitrogen, Carlsbad, CA) and analyzed on the Agilent Nanochip (Agilent Technologies, Santa Clara, CA). RNA samples were required to have a RNA Integrity Number (RIN) of 8 or greater to proceed with library creation. The RNA-Seq library was created using the mRNA Seq library preparation kit per manufacturer’s instructions (Illumina Inc., San Diego, CA). The library products are ready for sequencing via Illumina HiSeqTM 2000 and Sequencing was conducted in single-end reads. Above 30 million reads (about 200 bp insert size) clean data were recorded for each sample. Average and individual reads had Phred (passRead) scores of above 30.

### Analysis of gene expression

The sequences were mapped to the mouse reference gene and genome and counted. The gene expression level was calculated by using the RPKM method [52]. Differentially expressed genes were founded with the NOIseq method with the filtering condition of fold change≥2 and Probability≥0.8 [53].

### Data analysis

The synergistic antimicrobial effect of MEM and Amp combination treatment was analyzed using the CalcuSyn Software (Biosoft) [54]. For the analysis of the *in vitro* data, ANOVA and covariates followed by a multiple comparison test such as the Newmann-Keuls test were used to determine the statistical significance between the control and treatment groups. Software GraphPad Prsim 5.0 was used for analysis of data from animal experiments. *P*<0.05 was considered to be significant. All relevant data are within the paper and its Supporting Information files.

### Database

The protein access codes in Swissprot database are listed as follows: α7 nAChR, *Mus muscularus*, Q9JHD6; CD146, *Mus muscularus*, Q8R2Y2; CISH, *Mus muscularus*, Q62225; Irs1, *Mus muscularus*, P35569; IL-33, *Mus muscularus*, Q8bvz5; IL-18rap, *Mus muscularus*, Q9Z2B1; MfSD2a, *Mus muscularus*, Q9DA75; MMP9, *Mus muscular*, P41245,; MMP10, *Mus muscularus*,O55123; Zfp36, *Mus muscular*, P22893; Ptgds, *Mus muscular*, O09114; Tnfaip3 (A20), *Mus muscularus*, Q60769.
